# High Growth Potential of Long-Term Starved Deep Ocean Opportunistic Heterotrophic Bacteria

**DOI:** 10.3389/fmicb.2019.00760

**Published:** 2019-04-10

**Authors:** Marta Sebastián, Margarita Estrany, Clara Ruiz-González, Irene Forn, Maria Montserrat Sala, Josep M. Gasol, Celia Marrasé

**Affiliations:** ^1^Instituto de Oceanografía y Cambio Global, IOCAG, Universidad de Las Palmas de Gran Canaria, ULPGC, Gran Canaria, Spain; ^2^Departament de Biologia Marina i Oceanografia, Institut de Ciències del Mar, CSIC, Barcelona, Spain; ^3^Centre for Marine Ecosystems Research, School of Science, Edith Cowan University, Joondalup, WA, Australia

**Keywords:** long-term starvation, microbial seed bank, prokaryotes, bathypelagic, deep ocean, bacteria

## Abstract

Experiments with bacteria in culture have shown that they often display “feast and famine” strategies that allow them to respond with fast growth upon pulses in resource availability, and enter a growth-arrest state when resources are limiting. Although feast responses have been observed in natural communities upon enrichment, it is unknown whether this blooming ability is maintained after long periods of starvation, particularly in systems that are energy limited like the bathypelagic ocean. Here we combined bulk and single-cell activity measurements with 16S rRNA gene amplicon sequencing to explore the response of a bathypelagic community, that had been starved for 1.6 years, to a sudden organic carbon supply. We observed a dramatic change in activity within 30 h, with leucine incorporation rates increasing over two orders of magnitude and the number of translationally active cells (mostly Gammaproteobacteria) increasing 4-fold. The feast response was driven by a single operational taxonomic unit (OTU) affiliated with the *Marinobacter* genus, which had remained rare during 7 months of starvation. Our work suggests that bathypelagic communities harbor a seed bank of highly persistent and resourceful “feast and famine” strategists that might disproportionally contribute to carbon fluxes through fast responses to occasional pulses of organic matter.

## Introduction

The bathypelagic realm, the layer of the ocean between 1000 and 4000 m depth, is one of the largest habitats on Earth, and contains three-quarters of the pelagic marine prokaryotic biomass (Arístegui et al., 2009). Yet, how prokaryotes grow and survive in this habitat is still largely unknown. Dissolved organic carbon compounds are scarce and mostly refractory in deep waters (Hansell et al., 2012), and sinking particles produced in the sunlit layer of the ocean have long been assumed to sustain most of deep ocean heterotrophic activity (Herndl and Reinthaler, 2013). However, these particles are largely remineralized in the mesopelagic (200–1000 m depth) and the carbon reaching the bathypelagic is often not sufficient to meet the energy demands of deep ocean heterotrophic prokaryotes, which also rely on organic carbon produced through chemoautotrophy (Reinthaler et al., 2010).

Despite the contribution of dark carbon fixation to the prokaryotic carbon demand can be significant (accounting for up to half the surface derived particulate organic carbon, Reinthaler et al., 2010), and increasing findings of mixotrophic metabolisms in the deep ocean (Swan et al., 2011; Tang et al., 2016; Landry et al., 2017), evidence (albeit indirect) suggests that most prokaryotic heterotrophic activity relies on the carbon solubilized from particles (Baltar et al., 2009; Bochdansky et al., 2010, 2016). However, the flux of these particles varies in intensity and frequency over temporal and spatial scales (Conte et al., 2001; Arístegui and Montero, 2005; Smith et al., 2018), and prokaryotes have to cope with the sporadic nature of these pulses of carbon. Studies with heterotrophic bacterial isolates have shown that depending on the availability of carbon and energy, they alternate intermittent periods of growth (“feast”) with non-growth (“famine”) (Kjelleberg et al., 1993), and it has been observed that some surface marine heterotrophic bacteria remain viable after famine periods of months to years (Novitsky and Morita, 1977; Nissen, 1987; Kjelleberg et al., 1993; Finkel, 2006; Cappello et al., 2008; Pedler et al., 2014). However, whether they can swiftly respond to sudden carbon inputs after long famine periods remains unknown. Given the sporadic nature of the carbon supply, deep ocean heterotrophic prokaryotes should be able of persisting as inactive or dormant (i.e., in an state of low metabolic activity (Kjelleberg et al., 1993) for long times, and when inactive, such taxa would comprise a bathypelagic microbial seed bank (*sensu* Lennon and Jones, 2011) that may ensure a quick response of communities to any sudden resource input.

In a previous study we subjected a Mediterranean bathypelagic community to long-term (1 year) absence of external carbon inputs and observed a continuous succession of active phylotypes driven by recruitment from the seed bank, likely fuelled by the introduction of some labile dissolved organic matter (DOM) through chemolithoautotrophy (Sebastián et al., 2018). Whereas this demonstrated an astonishing capacity of bathypelagic communities to persist and survive under severe carbon deprivation, heterotrophic activity was very low throughout the experiment once the labile carbon had been consumed. Thus, it remains unknown if bathypelagic heterotrophic prokaryotes maintain their capacity to rapidly respond to fresh nutrient inputs after long periods of starvation.

Here we followed up on that previous experiment and used a multifaceted approach combining 16S rRNA gene sequencing, bulk activity measurements, and Catalyzed Reporter Deposition Fluorescent *in situ* Hybridization (CARDFISH) coupled with the single-cell activity technique Bioorthogonal non-canonical amino acid tagging (BONCAT), to study the timing and magnitude of the response of a carbon-deprived deep ocean community to the addition of freshly produced DOM.

## Results

**FIGURE 1:**
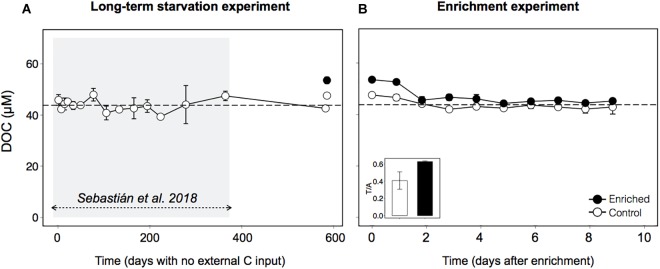
Dynamics of Dissolved Organic Carbon (DOC) in bathypelagic water in **(A)** the long-term starvation experiment (582 days with no external organic C input), and **(B)** the enrichment experiment, in which freshly produced DOM was added through mixing 4:1 with 0.2 μm filtered surface seawater (see section “Experimental Procedures”for details). Inset: ratio between peak T of fluorescent DOM, indicator of the amount of labile compounds, and peak A, indicator of the amount of humic compounds. The shaded area indicates the period studied in Sebastián et al. (2018). The data are presented as averages and standard deviations of three technical replicates for the long-term starvation experiment and three biological replicates for the enrichment experiment.

Organic carbon was supplied by mixing 4 parts of bathypelagic water with 1 part of 0.2 μm-filtered freshly collected surface seawater (see section “Experimental Procedures”), which represented an increase in the dissolved organic carbon concentration (DOC) of 9 μM over the ∼44 μM that had remained constant during the 1.6 years (Figure 1A,B). As a control, we used bathypelagic waters mixed in a 4:1 proportion with the same bathypelagic water filtered by 0.2 μm, to account for the dilution effect in the Enriched treatment. Manipulation of the Control treatment also led to some increase in the amount of organic carbon, as typically occurs after filtration (e.g., Hansell and Peltzer, 1998). However, after the long period with no external carbon inputs, this DOC should be less labile than the surface-derived DOC. To confirm this hypothesis, we examined the fluorescence characteristics of the DOC (Coble, 1996) in both treatments at the beginning of the experiment, and found that indeed the Control treatment had a higher proportion of recalcitrant humic-like substances (Peak A, see section “Experimental Procedures”) than labile protein-like substances (Peak T) (Figure 1B, inset).

**FIGURE 2:**
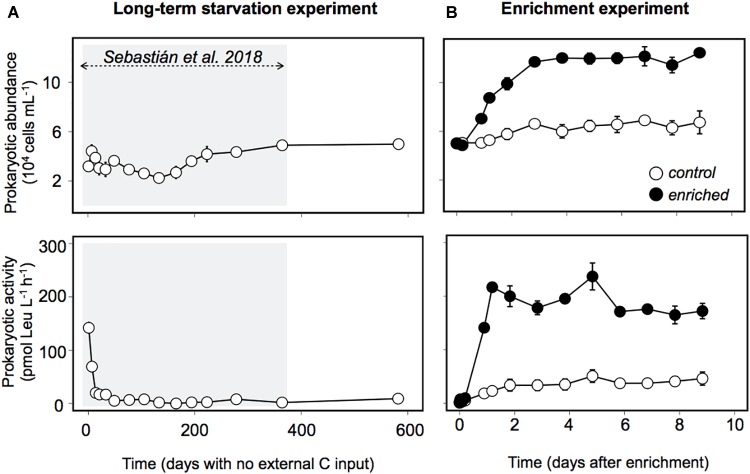
Prokaryotic abundance and activity (measured as ^3^H-leucine incorporation) during **(A)** the long-term starvation experiment and **(B)** the enrichment experiment. Black dots represent the Enriched treatment, white dots, the Control treatment. The data are presented as average and standard deviation of three technical replicates for the long-term starvation experiment and three biological replicates for the enrichment experiment. The shaded area indicates the period studied in Sebastián et al. (2018).

**FIGURE 3:**
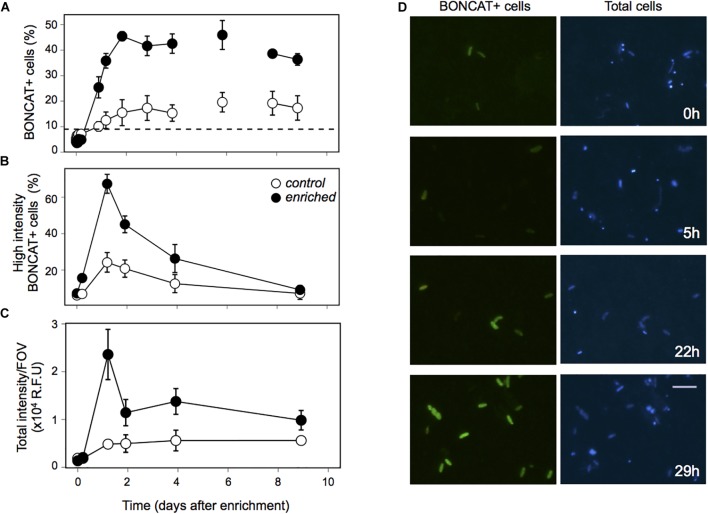
Single-cell activity estimated through Bioorthogonal non-canonical amino acid tagging (BONCAT) in the Enrichment experiment. **(A)** Percentage of translationally active cells (BONCAT+) in the Control (white dots) and Enriched (black dots) treatments. Dashed line indicates the percentage of BONCAT+ cells right before the Enrichment experiment was performed. **(B)** Percentage of high activity cells (within the top-third of BONCAT fluorescent signal), **(C)** Sum of the individual intensity of all BONCAT+ cells divided by the number of images considered for the image analysis. **(D)** Micrographs of the BONCAT+ cells in the Enriched treatment, showing how cells got activated during the first 29 h of the experiment. Left panel: BONCAT+ cells, Right panel: DAPI stained cells. Each data point in the graphs represents the average of three replicates, error bars represent the standard deviation.

**FIGURE 4:**
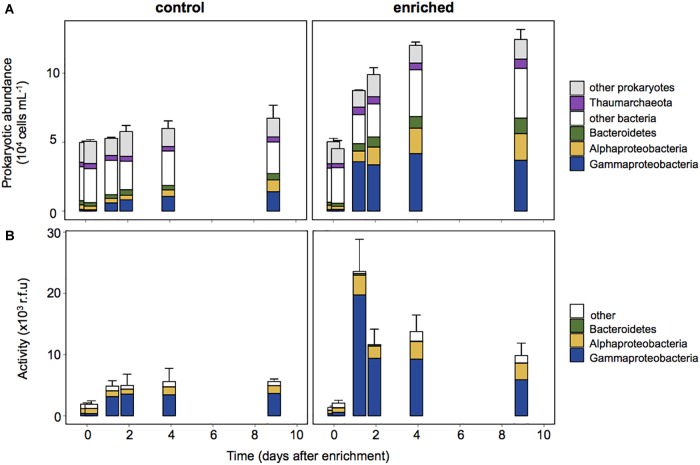
Prokaryotic abundance estimated by CARDFISH **(A)** and contribution to the activity of each phylogenetic group (**B**), estimated as the sum of the BONCAT signal intensity of cells belonging to each CARDFISH+ group in relation to the sum of the intensity of all BONCAT+ cells. Each data point in the graphs represents the average of three replicates, error bars represent the standard deviation for the total abundance.

**FIGURE 5:**
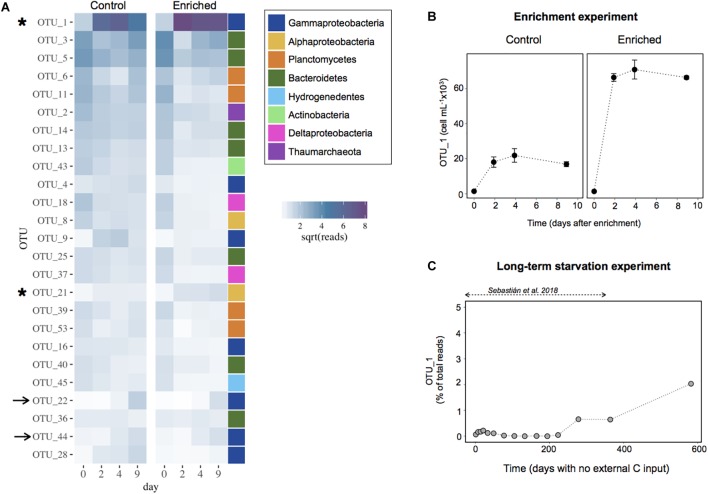
**(A)** Heatmap with the 25 most abundant OTUs in the Enrichment experiment. Data are shown as the relative contribution (in terms of sequences) assigned to each OTU. The values have been square root transformed for visualization. The color bar indicates the broad taxonomic categorization of each of the OTUs (at the class level for Proteobacteria, and phylum level for the rest). The arrows indicate the OTUs that increased similarly in proportion in both treatments, the asterisks those OTUs that experience a higher increase in the Enriched treatment. **(B)** Cell abundance attributed to the most responsive OTU (OTU1, affiliated to the *Marinobacter* genus) in the Enrichment experiment. The data are presented as means and standard deviation of 3 replicates. **(C)** Proportion of the community represented by the most responsive OTU1 during the long-term starvation experiment. The data are presented in relative contribution to total reads, to illustrate that this OTU was rare throughout most of the experiment.

Prokaryotic abundance remarkably increased within the first 2 days after enrichment, reaching later a plateau, with cell numbers that doubled the ones observed in the Control and those observed during the long-term starvation experiment (Figure 2A). The fact that prokaryotic abundance did not decrease with time suggests grazers and/or viruses did not play a significant role in controlling prokaryotic abundance in this experiment. Heterotrophic prokaryotic activity, estimated as leucine incorporation, increased two orders of magnitude within the first day in the Enriched treatment, and remained high throughout the experiment (Figure 2B). Activity also increased in the Control treatment, probably driven by the increase in DOC and some relief in the competition for resources due to the dilution with 0.2 μm filtered water. However, this increase in bulk activity was 4-fold lower than that in the “Enriched” treatment (Figure 2B) and 3 to 5 times lower when specific activities were considered (Supplementary Figure 1). Single-cell analyses of translational activity were performed by the technique BONCAT (Dieterich et al., 2006; Hatzenpichler et al., 2014) which allows a fast and sensitive characterization of individual cell translational activity through their fluorescence signal intensity (Leizeaga et al., 2017). After 1.6 year of no external input of carbon, only 10% of the cells were translationally active, or with enough activity to be detected within the incubation time (Figure 3A, dashed line). Upon manipulation, the number of BONCAT+ cells increased in both the Enriched and the Control treatments but the increase was 4-fold higher in the Enriched treatment (Figure 3A,D). This boost can be explained by increased DOC availability, but also by the higher lability of the added organic matter (Figure 1B, inset), as reflected also by the rise in activity of the enzymes devoted to the usage of labile carbon compounds (Supplementary Figure 2). Although the percentage of active cells in the Enriched treatment remained high throughout the experiment, we observed a major drop in single-cell translational activity after the first day, as shown by the decrease in the proportion of cells with high activity and the total fluorescence intensity of the BONCAT signal (Figure 3B,C). Despite this drop, the pool of active cells in the Enriched treatment was dominated by cells with high or medium activity from day 1 until day 9, when cells with low activity represented 50% of the cells (Supplementary Figure 3). In contrast, most of the active assemblage in the Control treatment displayed low activity throughout the experiment. This suggests that in the Enriched treatment, cells showed a swift feast response upon the pulse of labile DOM, but then activity levels dropped after 1 day, likely due to the usage of most of the readily available DOM. Despite this drop in individual cell activity, bulk prokaryotic activity remained fairly constant in the Enriched treatment due to the increase in cell abundances (Figure 2B). The fact that on day 9 half of the active pool in the Enriched treatment corresponded to low activity cells (Supplementary Figure 3) may indicate that by then most of the labile DOM had been consumed.

To investigate which members of the prokaryotic assemblage drove the changes observed in activity, we combined BONCAT with CARDFISH. Gammaproteobacteria cell abundance (detected by CARDFISH) increased in both the Control and the Enriched treatment. They represented only 2% of the cells at the beginning of the experiment, but accounted for up to ∼25% and ∼40% in the Control and Enriched treatments, respectively (Figure 4A), showing a notable increase in abundance within 29 h after enrichment. Other phylogenetic groups, like Alphaproteobacteria and Bacteroidetes, also increased their relative abundance upon enrichment from 6 to 13%, and from 4 to 8%, respectively. BONCAT coupled with CARDFISH indicated that the increase in activity in both treatments was due to Gammaproteobacteria (Figure 4B), which accounted for up to 84% of the activity on day 1 (contribution to the BONCAT intensity signal, see section “Experimental Procedures”) in the Enriched treatment and 71% of the activity in the Control treatment (from day 2 to 9, Figure 4B).

We next explored the dynamics of the most abundant OTUs in both the Enriched and the Control treatments to identify the individual taxa driving the response to enrichment (Figure 5A). Surprisingly, the abundant OTUs displayed very similar dynamics in both treatments, and the response seemed to be largely due a single OTU (OTU1) affiliated to the genus *Marinobacter* within the Alteromonadales order (Supplementary Table S1). Within 2 days, this OTU represented ∼30 and 65% of the sequences in the Control and Enriched treatment, respectively. Only a few other OTUs increased slightly upon enrichment but in general they showed a similar increase in both treatments, except for OTU21, affiliated to the Alphaproteobacteria (Figure 5A and Supplementary Table S1). Despite the *Marinobacter* OTU1 represented a large percentage of the community in the Control, the cell abundances of this OTU were estimated to be three times higher in the Enriched treatment than in the Control (Figure 5B).

We then looked at the dynamics of this responsive OTU during the long-term starvation experiment (Figure 5C). Interestingly, the *Marinobacter* OTU was rare (0.07% of the reads) at the beginning of the starvation period and remained rare or undetected for 7 months (∼200 days). Afterward, this OTU increased in abundance, reaching 2% of the reads 3 days before starting the Enrichment experiment, 1.6 years after water collection (day 582). This suggests that besides being the most successful bloomer upon fresh carbon addition, this persistent OTU was also able to cope for a long period with no external carbon input.

## Discussion

Conditions that allow sustained growth are rare in nature, and some heterotrophic bacteria adapt to this situation by alternating periods of growth with non-growth (Kjelleberg et al., 1993). Although experiments with isolates have shown that they may persist from months to years in the non-growth state, persistence and feast responses have been rarely explored in natural communities, where there is a high level of diversity and non-growing cells may be overridden by better competitors.

Here we show a remarkable capacity of long-term carbon deprived deep ocean heterotrophic bacteria to activate and grow upon a sudden pulse in resource availability. After a famine period of ∼1.6 years, a relatively moderate addition of labile carbon led to a fast rise in prokaryotic heterotrophic activity, which increased two orders of magnitude in only 1 day. This was followed by an increase in prokaryote abundances, which in 2 days doubled the values observed before enrichment. Manipulation during the experimental set-up (i.e., filtration) also led to a small increase in both activity and abundance in the Control treatment due to a rise in DOC concentrations and some relief in the competition for resources after the dilution with filtered water. However, the magnitude of this response in terms of activity was 4-fold lower than in the Enriched treatment, likely due to both the lower quantity and lability characteristics of the DOC. Nevertheless, it seems that the difference in DOM characteristics was not sufficient to promote changes in community composition, because the 25 dominant taxa were shared among both treatments and generally showed similar relative abundances (Figure 5A). The few OTUs that increased in abundance in the Enriched treatment, displayed an increase in the Control, suggesting they could also take advantage of the slight enrichment produced in the Control. Yet, the response observed was clearly driven by the *Marinobacter* OTU1, which bloomed in the Enriched treatment (Figure 5A,B).

It could be argued that the response we observed in the Enriched treatment was driven by bacteria that passed through the 0.2 μm pore size filter of the surface waters we used for enrichment. Yet, the fact that the same *Marinobacter* OTU grew in both the Control and Enriched treatments provides evidence against this argument. Nevertheless, we estimated the putative contribution of surface derived *Marinobacter* to the initial pool of *Marinobacter* cells and found that they could only represent 1.5% of the *Marinobacter*, and 2.7% of the active Gammaproteobacterial pool (see Supplementary Figure 4 and Supplementary Table S2 for further details). Given the growth rates displayed by the Gammaproteobacterial cells in our experiment [0.11 h^-1^, calculated from the CARDFISH counts as in Ferrera et al. (2011)], it is not possible that surface derived cells accounted for the swift response we observed upon enrichment.

The bathypelagic realm can be seen as a large oceanic desert with intermittent pulses of labile carbon introduced through surface-derived sinking particles (Conte et al., 2001; Smith et al., 2018) and a myriad of diverse compounds that are either recalcitrant or difficult to digest (Jiao et al., 2010) or so diluted that are below the threshold needed to invest in the necessary machinery for their uptake and degradation (Arrieta et al., 2015). The imprint of the pressure for survival in such a carbon-limited system likely explains the prevalence of mixotrophic and autotrophic metabolisms, unveiled by genomic studies and single-cell techniques (Herndl et al., 2005; Swan et al., 2011; Sheik et al., 2014; Li et al., 2015; Landry et al., 2017; Pachiadaki et al., 2017). It is likely that bathypelagic heterotrophic bacteria efficiently exploit resource patches when associated to particles, but also are able to persist between successful encounters with particles, either by becoming inactive, or by using recalcitrant compounds or alternative sources of energy, or both. These bacteria can be considered “opportunitrophs” (*sensu* Moran et al., 2004), ready to take immediate profit of any of the scarce opportunities to feed and grow.

**FIGURE 6:**
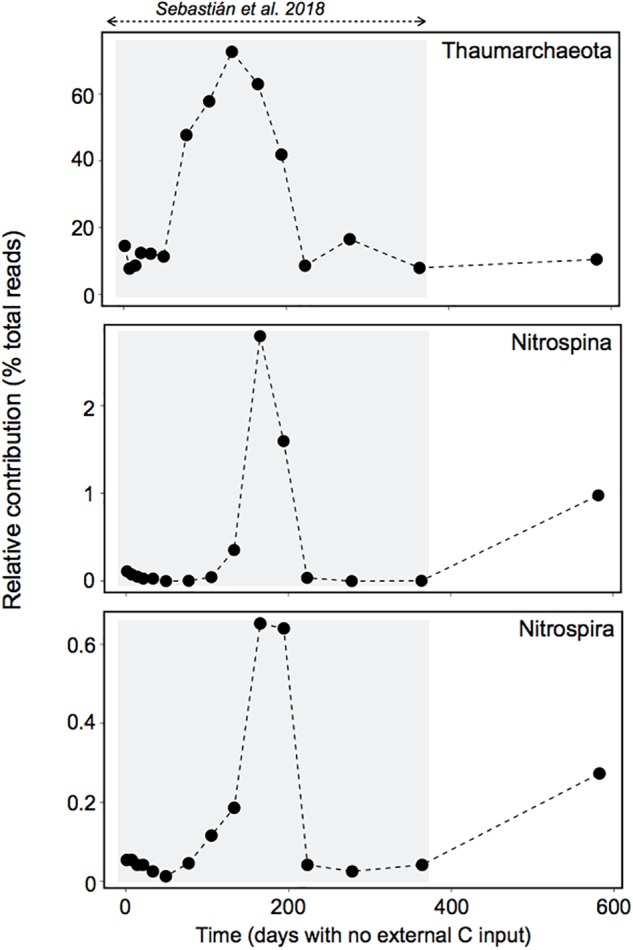
Dynamics of potential chemolithoautotrophs during the long-term experiment. The gray box indicates the period studied in Sebastián et al. (2018). The abundance of these groups peaked right before the activation of the dominant *Marinobacter* OTU, and they also increased in abundance during the last 6 months of starvation, before the Enrichment experiment was performed.

Our results suggest that a single *Marinobacter* OTU belonging to the Alteromonadales order was responsible for most of the observed response in activity. This OTU attained ∼65% of the total Enriched community sequences in less than 1 day (Figure 5A). Members of the genus *Marinobacter* have been described as opportunistic, with plasticity to utilize a broad spectrum of substrates, including recalcitrant carbon compounds such as lipids, hydrocarbons and aromatic compounds (Kostka et al., 2011; Handley and Lloyd, 2013), and various terminal electron acceptors, to which the cell can rapidly adjust in response to changing environmental conditions (Singer et al., 2011). Our findings support the view of *Marinobacter* bacteria as opportunitrophs, because besides being able to quickly exploit fresh surface-derived organic matter, the most responsive OTU also managed to cope with a long-term absence of external inputs of organic carbon probably from the slow reworking of recalcitrant compounds and/or the input of some labile carbon through chemolithoautotrophy (see Sebastián et al., 2018). The later is supported by the observation that typical chemolithoautotrophs like Thaumarchaeota, Nitrospina and Nitrospira showed a peak of abundance right before the activation of the *Marinobacter* OTU, 6–7 months after the start of the long-term experiment (Figure 6), with Thaumarchaeota representing ∼70% of the 16S-based community. A recent study with different Thaumarchaeota isolates has shown that they release nitrogen-rich labile organic compounds, such as amino acids, thymidine and vitamins, that fuel heterotrophic bacteria, and the release of these compounds follows the growth of the different Thaumarchaeota strains, as indicated by nitrite production (Bayer et al., 2019). In our experiment we also observed a peak of nitrite coincident with the rise in Thaumarchaeota relative abundance (Supplementary Figure 5 and Figure 6), which supports our hypothesis that chemolithoautotrophs supplied complementary energy for the long-term persistence of the *Marinobacter* OTU during the starvation experiment, as well as for the maintenance of the heterotrophic community (Sebastián et al., 2018). Noteworthy, Nitrospina and Nitrospira also increased in abundance during the last 6 months of the long-term starvation experiment, before the Enrichment experiment was performed (Figure 6), coinciding with the rise in the relative abundance of the *Marinobacter* OTU (Figure 5C). Although the abundance of Nitrospina was generally low (>3%), they may have played an important role in carbon fixation because they are much larger than Thaumarchaeota in terms of biomass, and significantly contribute to carbon fixation in dark waters (Pachiadaki et al., 2017). The fact that both Nitrospina and Nitrospira increased in abundance at the end of the long-term experiment suggests that they may have provided the necessary energy boost for the increase in abundance of the *Marinobacter* OTU before the enrichment (Figure 5C), as well as for its fast response upon DOM supply (Figure 5B).

A search for *Marinobacter* OTUs in a global ocean dataset (Mestre et al., 2018), revealed that despite their generally low abundances, these bacteria are ubiquitous, and are present throughout the water column down to 4000 m depth both in the free-living and particle associated fractions (Supplementary Figure 6). In particular, our successful bloomer (*Marinobacter* OTU1) was found to be a quite widespread member of the marine rare biosphere, present in very low abundances in both the sunlit and dark layers of the ocean and across different basins (Mediterranean, South Atlantic, North Pacific). In view of the reported combination of persistence, and capacity to rapidly react to sudden resource availability, it is thus possible that the *Marinobacter* genus is a relevant member of the bathypelagic microbial seed bank. Although the existence of an oceanic seed bank was proposed several years ago (Caporaso et al., 2012; Gibbons et al., 2013) to our knowledge this is the first evidence that rare marine taxa within complex natural communities can persist for such a long time and quickly reactivate, contributing disproportionally to carbon cycling.

In summary, our results support a great ability of bathypelagic communities to persist and maintain functionality upon prolonged carbon scarcity, and shed light on successful and extremely plastic feast and famine strategies within some rare deep ocean opportunitrophs. We show that these opportunitrophs can grow fast and display sudden peaks of activity even after long periods of carbon deprivation, and thus they may play a significant role in carbon processing in the bathypelagic desert. Our work also provides further proof of the role of the seed bank in maintaining the diversity and ensuring the long-term function of microbial communities.

## Experimental Procedures

### Experimental Set-Up

The bathypelagic water (600 L) had been collected from 2100 m in the Northwestern Mediterranean Sea (40° 38′ 31.01″N, 2° 51′ 1.6″W) in October 2014 during the MIFASOL-I cruise from on board the R/V García del Cid. All the details about how the long term starvation experiment was performed can be found in Sebastián et al. (2018). Briefly, bathypelagic water was introduced in a 600-L container and maintained in the dark and at ∼17°C, which is only 4°C higher than the typical bathypelagic Mediterranean Sea temperature (Luna et al., 2012). The container was airtight except for the moment of sampling, so a certain gas exchange was allowed. Prior to each sampling, the container was gently agitated by moving it back and forth using a pallet jack and samples were siphoned out of the tank by means of an autoclaved and acid-rinsed glass-tube attached to a long silicon tube, with the help of a 60-mL syringe. The lid of the container covered the tube during the sampling, to avoid immigration of foreign taxa. Samples were taken at different time intervals [on days 1, 7, 14, 21, 33, 49, 77, 105, 133, 165, 194, 223, 278, 364 (Sebastián et al., 2018)]. For the present study, an additional sample was collected on day 582, right before the setup of the carbon addition experiment (hereafter the Enrichment experiment) to characterize the community prior to the experimental manipulation. Water for the Enrichment experiment was collected on day 585, and the experiment was started that same day.

The Enrichment experiment was performed in 20 L containers that had been thoroughly acid washed, and rinsed 3 times with milliQ and with the sampling water. Surface water for the enrichment was collected from the Blanes Bay Microbial Observatory (BBMO, Mediterranean Sea 41°40’N, 2°48’E) and the microbial biomass was removed by filtration through a 0.2 μm pore size polycarbonate filter. Sixteen-L of bathypelagic water from the container (which had had no external input of carbon for 1.6 years) was dispensed into six 20-L containers. Three of these containers were amended with 4 L of the 0.2 μm-filtered freshly collected surface seawater (“Enriched treatment”). The “Control treatment” (the other three containers) consisted on adding 4 L of the same bathypelagic water but 0.2 μm-filtered right before the addition, to account for the dilution effect experienced in the Enriched treatment. We performed this control because dilution with filtered water causes a certain relief in the competition for resources, and we wanted to address the differences between the effect of the dilution and the effect of enrichment with more labile surface-derived carbon. The containers were kept in the dark and at ∼17°C during the experiment.

Enrichment of bathypelagic waters with surface waters may not seem ideal, but we wanted to enrich the starved water with naturally occurring compounds, and sampling fresh bathypelagic waters was not feasible for logistics reasons. Nevertheless, deep water enrichment with surface waters cannot be considered totally artificial, since sinking of surface DOM is quite frequent in the Mediterranean Sea during deep water formation events (Santinelli, 2015).

Samples were taken several times within the first day, and on a daily basis during the next 9 days. Since we are aware that filtration is not 100% efficient in removing bacterial cells, we also took samples from the filtrate for DNA (5 L) and bacterial abundance to check for potential contamination with surface taxa. Less than 1% of the cells passed the 0.2 μm-filter.

### Prokaryotic Abundance and Heterotrophic Production

Prokaryotic cell abundance was estimated by flow cytometry as described elsewhere (Gasol and Morán, 2015). Potential prokaryotic heterotrophic production was estimated from the incorporation of tritium-labeled leucine (Kirchman et al., 1985) using the centrifugation procedure. Four replicates of 1.2 ml and two trichloroacetic acid (TCA)-killed controls were incubated with ^3^H-Leucine at a final concentration of 40 nM. Incubation was performed in the dark at *in situ* temperature for 4 h and stopped with 5% TCA, final concentration. The samples were then kept frozen at -20°C. Once thawed, they were processed as described elsewhere (Smith and Azam, 1992).

### Single-Cell Activity

Single-cell activity was estimated using BONCAT (Dieterich et al., 2006) following the protocol detailed in Leizeaga et al. (2017). 50 mL samples were incubated with L-homopropargylglycine (HPG) (100 nM final concentration). This volume was reduced to 25 mL for the Enriched treatment after 2 days, due to the increase in prokaryotic abundance. Preliminary tests were run before the “Enrichment” experiment to decide an appropriate incubation time: enough to detect active cells in the Control, but short enough to be able to sample several time points just after the enrichment to monitor the short-term response. These tests indicated that for an incubation time between 30 min and 6 h the percentage of BONCAT-positive cells (hereafter BONCAT+) was rather constant in the long-term starved bathypelagic water (∼10%), so we proceeded with 30 min for all the BONCAT incubations. After the incubation, samples were fixed with 0.2 μm-filtered paraformaldehyde [PFA, final concentration 1% (v/v)] overnight at 4°C. The samples were then gently filtered through a 0.2 μm pore size polycarbonate filter, washed three times with sterile milliQ water, and kept frozen at -80°C until further processing. After thawing, the filters were dipped in 0.1% low-gelling-point agarose, dried at 37°C, and later dehydrated with 95% ethanol. This allowed attachment of the cells to the filters to prevent cell loss during permeabilization and downstream procedures. Cell walls were permeabilized with lysozyme (10 mg ml^-1^; 0.05 M EDTA, 0.1 M Tris–Hcl, 1 h) and achromopeptidase (60 U ml^-1^, 0.01 M Na Cl, 0.01 M Tris–Hcl, pH 7.6, 30 min) at 37°C following standard protocols (Sekar et al., 2003). Each filter was cut into either 1/10 or 1/6 slices using a sterile razor blade. The remaining portion was stored (-80°C). Cu(I)-catalyzed click chemistry was later performed, as detailed below.

For the click reaction, a dye-premix was prepared by mixing 10 μl of 20 mM of filter-sterilized CuSO_4_, 20 μl of 50 mM of filter-sterilized Tris[(1-hydroxypropyl-1H-1,2,3-triazol-4-yl)methyl]amine (THPTA^1^) and 8 μl of 1 mM of the CR110 azide dye. This premix was allowed to react for 3 min at room temperature in the dark. In the meantime, 100 μl of a freshly prepared 100 mM solution of sodium ascorbate and 100 μl of a freshly prepared 100 mM solution of aminoguanidine hydrochloride were added to 1.7 mL of phosphate buffered saline (PBS) solution. Then, for the click reaction mix, the dye-premix was added to the PBS-ascorbate-aminoguanidine mix and the tube was inverted twice. The portions of filters were then placed on an empty 1.5 mL eppendorf tube and the reaction mix was added to it, including to the cap to further avoid any air bubbles. The eppendorf tube was closed and incubated in the dark at room temperature for 30 min. After the click-reaction, the filters were washed three times for 3 min each in PBS-filled petri dishes and finally they were dehydrated by incubating them for 3 min in increasing concentrations of ethanol: 50%, 70%, 96%, at RT. Filters were counterstained with 4′,6-diamidino-2-phenylindole (DAPI, 10 μg mL^-1^ final concentration) and analyzed by epifluorescence microscopy.

Killed controls (samples fixed before the HPG addition) were included with all sets of samples to correct for background fluorescence from naturally occurring azides.

Images were acquired using an Axio Imager.Z2m epifluorescence microscope connected to a Zeiss camera (AxioCam MRm, Carl Zeiss Microscopy, Germany) at 63× magnification through the Axiovision software, and analyzed using ACMEtool, as detailed in Leizeaga et al. (2017). Detailed information about how the images were acquired and how the downstream analyses were performed can be found in the Supplementary methods.

The intensity of the BONCAT+ cells was assessed using the mean gray value (MGV), which is the sum of the gray values of all the pixels in the cell divided by the number of pixels. The intensities of individual cells were rank-ordered to obtain the maximum and minimum values and the intensity range was then equally divided into three groups: high intensity (top third), intermediate intensity (middle third) and low intensity cells (bottom third). The percentage of each intensity group within the BONCAT+ cells was calculated at each time point, for each sample. We found that the minimum MGV in all samples was 24–30, and the maximum 224–230, so all samples were divided in three similar groups of fluorescence intensity, and thus we could compare the results across samples.

## Catalyzed Reported Deposition Fluorescence *in situ* Hybridization (CARDFISH)

CARDFISH was performed to address changes in community composition following the protocol detailed in Pernthaler et al. (2004). For the quantification of Thaumarchaeota the cells were further permeabilized by dipping the filter portions in HCl 0.1 M for 1 min and then washed in PBS.

The filters were hybridized with one of the following horseradish peroxidase (HRP)-labeled probes: EUB338 I-II and –III (targets most Bacteria, Daims et al. (1999), GAM42a together with its unlabeled competitor probe (targets most Gammaproteobacteria, Manz et al. (1992), CF319a and CF968 (targets many members of the Bacteroidetes group, Manz et al., 1996; Acinas et al., 2015), Alf968 (targets most Alphaproteobacteria, Neef, 1997) and Cren554 (targets Thaumarchaeota, Massana et al., 1997). Specific hybridization conditions were established by addition of formamide to the hybridization buffers (45% formamide for the Alf968 probe, 20% for Cren554 and 55% for the other probes). Hybridization was performed overnight at 35°C (Pernthaler et al., 2004). For amplification, we used tyramide labeled with Alexa 594.

Filters were cut and stained with 4′,6-diamidino-2-phenylindole (DAPI, final concentration 1 μg mL^-1^) to quantify the abundance of the different phylogenetic groups in relation to total prokaryotic counts.

At some of the time-points (0, 0.2, 1, 2, 4, 9 days) BONCAT was combined with CARDFISH to assess the contribution of different prokaryotic groups to community activity. For that purpose, BONCAT was performed after the tyramide amplification, following the protocol described above. Although it has been argued that the BONCAT fluorescence intensity of the cells may vary depending not only on the amount of proteins produced but also on the methionine content of these proteins (because HPG is a surrogate for methionine) (Hatzenpichler and Orphan, 2015), the fluorescence intensity is strongly correlated to leucine incorporation in marine planktonic samples (Leizeaga et al., 2017). Therefore, the contribution of each bacterioplankton group to the activity was calculated as the sum of the intensity of all the BONCAT+ cells belonging to each bacterioplankton group (CARDFISH+), divided by the sum of the intensity of all BONCAT+ cells.

### Total Organic Carbon

Ten-mL water samples were collected in precombusted (450°C, 24 h) glass ampoules. After adding 50 μL of 25% H_3_PO_4_ to acidify at pH <2, the ampoules were heat-sealed and stored in the dark at 4°C until analysis. TOC concentrations were quantified with a Shimadzu TOC-LCSV organic C/N analyser. Between 3 and 5 injections of 150 μL per replicate were performed and the final organic carbon concentration in each sample was calculated by subtracting a Milli-Q blank and dividing by the slope of daily made standard curves made from potassium hydrogen phthalate. Reference samples of the Material Reference Certificate (MRC Batch-13 Lot // 08-13, Hansell Laboratory. University of Miami, RSMAS) were used daily for quality control. Since particulate organic carbon represents less than 1% of the TOC in the deep ocean (Mopper and Qian, 2006), TOC can be considered equal to dissolved organic carbon (DOC) within the analytical precision of the method, and therefore we have used the term DOC throughout the paper.

Samples to analyze Fluorescent Dissolved Organic Matter (FDOM) were taken on day 0 of the Enrichment experiment. FDOM was measured using a Perkin Elmer LS55 luminescence spectrometer provided with a xenon discharge lamp equivalent to 20 kW for an 8-μs duration. A red sensitive R928 photodiode multiplier operated as a reference detector. Single measurements were performed in a 1 cm acid-cleaned quartz fluorescence cell at a constant room temperature. Fluorescence cells were rinsed with water sample before analyses. Following (Coble, 1996), the Ex/Em wavelengths used for the single point measurements were: Ex/Em 250 nm/435 nm (peak-A) and Ex/Em 280 nm/350 nm (peak-T) as indicators of humic and protein-like substances, respectively.

### Nucleic Acid Extraction

Samples for nucleic acid extraction (2-L) were taken at days 0, 2, 4, and 9. An extra sample (5-L) was collected from the 0.2 μm filtrate of the freshly collected surface seawater to confirm there was no immigration of surface-derived taxa that could mislead the results obtained. All samples were filtered onto a 0.2 μm polycarbonate membrane filtered and filters were stored frozen until extraction.

Total nucleic acid extractions were performed using the standard phenol-chloroform protocol with slight modifications (Logares et al., 2014). DNA was quantified using a Qubit fluorometer assay (Life Technologies, Paisley, United Kingdom). The V4–V5 region of the 16S rRNA gene was amplified with the primers 515F and 926R (Parada et al., 2016) and sequenced in an Illumina MiSeq platform using 2 × 250 bp paired-end approach at the Research and Testing Laboratory facility (Lubbock, Texas, United States^2^).

### Data Analyses

Computing analyses were run at the Marine Bioinformatics Service of the Institut de Ciències del Mar (ICM-CSIC) in Barcelona. Illumina (Inc., San Diego, CA, United States) sequences were quality filtered following (Logares, 2017). The obtained sequences were combined with those obtained during the long-term starvation (ENA ERS1993945–ERS1993972), clustered into operational taxonomic units (OTUs) at 99% cutoff using the UPARSE algorithm implemented in USEARCH (Edgar, 2013). Singletons (i.e., OTUs occurring once in just one sample) and chimeric OTUs were removed as described elsewhere (Logares, 2017). The remaining OTUs were taxonomically annotated using BLAST against the SILVA128 database as reference. OTUs assigned to chloroplasts were removed from subsequent analyses. All raw sequences used in this study are publicly available at the European Nucleotide Archive (ERX3017327–ERX3017349).

Data treatment and statistical analyses were performed with the R (version 3.3.2) and RStudio softwares (version 1.0.44). The OTU table was randomly subsampled to the lowest number of reads per sample in order to avoid statistical artifacts due to an uneven sequencing effort among samples. For this we used the *rrarefy* function in the *vegan* package (Oksanen et al., 2015) and one of the replicates of day 9 of sampling was excluded due to the low number of reads as it contained (5,425 as compared to a range in the number of reads per sample of 24,212–151,518). Cell abundance of the OTUs was estimated by multiplying the relative proportion of 16S rDNA reads by the prokaryotic cell abundance, as in Alonso-Sáez et al. (2015).

## Author Contributions

MS and CM designed the experiments. MS analyzed the data and wrote the manuscript. ME performed the single-cell analyses. CR-G carried out the prokaryotic activity measurements. IF helped with the image analyses. All authors made comments and relevant editions to the text.

## Conflict of Interest Statement

The authors declare that the research was conducted in the absence of any commercial or financial relationships that could be construed as a potential conflict of interest.

## Footnotes

1 www.clickchemistrytools.com

2 http://www.researchandtesting.com
