# Supplementary material for: High Growth Potential of Long-Term Starved Deep Ocean Opportunistic Heterotrophic Bacteria
Source: Front Microbiol. 2019 Apr 10;10:760. doi: 10.3389/fmicb.2019.00760 (PMC6468046; doi:10.3389/fmicb.2019.00760)
Supplement: Supplementary file 2 [file Data_Sheet_1.PDF]

SUPPLEMENTARY INFORMATION

High growth potential of long-term starved deep ocean opportunistic heterotrophic bacteria

Marta Sebastián, Margarita Estrany, Clara Ruiz-González, Irene Forn, M. Montserrat Sala, Josep M. Gasol, Celia Marrasé

This file contains **supplementary methods** and the following **supplementary figures and tables**:

Supplementary figure 1. Temporal dynamics of cell specific heterotrophic prokaryotic production in the control (white dots) and enriched (black dots) treatments of the enrichment experiment. Values represent the average and standard deviation of three replicates.

Supplementary figure 2. Temporal dynamics of hydrolytic enzymes involved in the degradation of carbohydrates (ß-glucosidase) and peptides (leu-aminopeptidase) in the control (yellow dots) and enriched (green dots) treatments of the enrichment experiment. Values represent the average and standard deviation of three replicates.

Supplementary figure 3. Percentage of cells with low, medium and high activity in the enrichment experiment, estimated based on the fluorescent intensity of the BONCAT signal of the individual cells. The intensity of the BONCAT+ cells was assessed using the mean gray value, which is the sum of the gray values of all the pixels in the cell divided by the number of pixels. The intensities of individual cells were rank-ordered to obtain the maximum and minimum values and the intensity range was then equally divided into three groups: high intensity (top third), intermediate intensity (middle third) and low intensity cells (bottom third). The percentage of each intensity group within the BONCAT+ cells was then calculated at each time point.

Supplementary figure 4. Comparison of the relative abundance of gammaproteobacterial cells estimated by CARDFISH and *Marinobacter* OTU sequences expressed as percentage of total cells, and reads, respectively.

Supplementary figure 5. Dynamics of nitrate and nitrite concentration during a) the long-term starvation experiment and b) the Enrichment experiment. Values represent the average and standard deviation of two technical replicates in the case of the Long-term experiment, and three replicates in the case of the Enrichment experiment. Nitrite displayed a peak coincident with the raise in ammonia oxidizers, but decreased drastically when nitrite oxidizers developed (see Figure 6 in the main text).

Supplementary figure 6. Contribution of *Marinobacter* OTUs to total sequences in a global oceanic survey (Malaspina 2010, Mestre et al. 2018). Upper panel: free-living communities (0.2-3µm), lower panel: particle associated communities (3-20µm). SFC: surface, DCM: Deep Chlorophyll Maximum depth, Meso: mesopelagic (200-1000m), Bathy: bathypelagic (1000-4000m). Asterisks denote those samples where the *Marinobacter* OTU that dominated in the Enrichment experiment was detected. The green asterisk represents the location where the water for the long-term starvation experiment was collected.

Table S1. Relative Abundance (mean and standard deviation) and taxonomic affiliation of the 25 most abundant OTUs in the Enrichment Experiment

Table S2. Evaluation of the potential contamination of the Enriched treatment with taxa present in the 0.2µm filtered surface seawater used for enrichment. Cell abundances of the *Marinobacter* OTU was calculated using the relationship shown in Figure S3, taking into account the relative abundance of the OTU (%reads) and the total abundance of prokaryotic cells (estimated by flow cytometry). Values are shown as average and standard deviation in parentheses (when possible). Even assuming that all surface derived *Marinobacter* OTU_1 cells were active, they only represented 2.7% of the total pool of

Gammaproteobacteria active cells. Growth rate of Gammaproteobacterial cells in the Enriched treatment —estimated by CARDFISH, as in Ferrera et al. (2011)— was 0.11 h^{-1}. Growing at this rate, the surface derived OTU would reach an abundance of 576 cell mL^{-1}, which is two orders of magnitude lower than the actual values. Thus, we can rule out that surface *Marinobacter* cells were the ones driving the response to enrichment.

SUPPLEMENTARY METHODS

Enzymatic assays

We monitored the extracellular enzyme activities of Leu-aminopeptidase, and β-glucosidase, using L-leucine-7-amino-4-methylcoumarin and 4-methylumbelliferyl β-D-glucoside as substrates, respectively (all purchased at Sigma-Aldrich) following the method developed by Hoppe (1983). Assays were performed following the modification described in Sala *et al.* (2016). Briefly, each sample (350 µl) was pipetted in quadriplicate into 96 black well plates, and substrates were added to obtain a final concentration of 125 µM. The fluorescence of the 96 well plates was measured at the beginning and after 3 hours incubation at in situ temperature conditions with a Tecan Infinite 200 microplate reader at 365 nm excitation and 450 nm emission wavelengths. The increase in fluorescence in each well was converted into activity using a standard curve prepared with the fluorophores 4-methylumbelliferone (MUF) or 4-methylcoumarinyl-7-amide 4 (Sigma-Aldrich).

Image analyses of BONCAT and CARDFISH micrographs

All images (at least 15 fields/filter and more than 3000 cells per portion of filter (range 3000-10000 cells) were acquired at: (i) 350 mn excitation, 457 nm emission (UV channel for DAPI) with 20 ms exposure time when taken with the black & white camera and with 100 ms with the colour camera (these exposures times were selected after several tests to guarantee all fluorescent cells were captured in the analyses), (ii) 490 nm excitation, 520 nm emission (blue channel for Cr110 azide) with 300 ms exposure time both in color and black and white, (iii) 590 nm excitation, 620 nm emission (Green channel for Alexa 594) with a wide range of exposure times depending on the analysed taxonomic group (from 200 to 2000 ms) to ensure we considered all the cells belonging to that group.

For BONCAT analyses, pictures were taken in black and white, two micrographs from the same field of view, one for DAPI and another for BONCAT. The percentage of BONCAT+ cells

was calculated in relation to the DAPI counts. ACMEtool was used for subtracting the background of the samples. For that purpose, the signal-to-background ratio of the cells was adjusted until the killed control displayed 0-1.5% of BONCAT+ cells because of background noise observed from DAPI staining of the killed controls.

For BONCAT-CARDFISH analyses, pictures were taken in colour because the fluorochrome used for CARDFISH (Alexa594) also emitted some red fluorescence under the blue light used for detecting BONCAT labelled cells, and thus although both signals could be distinguished in a colour image (BONCAT+ cells in green and CARDFISH-positive (CARDFISH+) cells in red) they could not be differentiated in a black and white image. Since the ACMEtool software requires black and white images for the analyses, colour images were transformed to black and white images using the ImageJ program, which allows separation of different colours in different channels. Therefore, image analyses were performed over three black and white pictures of the same visual field: one for the DAPI stain, one for the BONCAT+ cells and one for the CARDFISH+ cells.

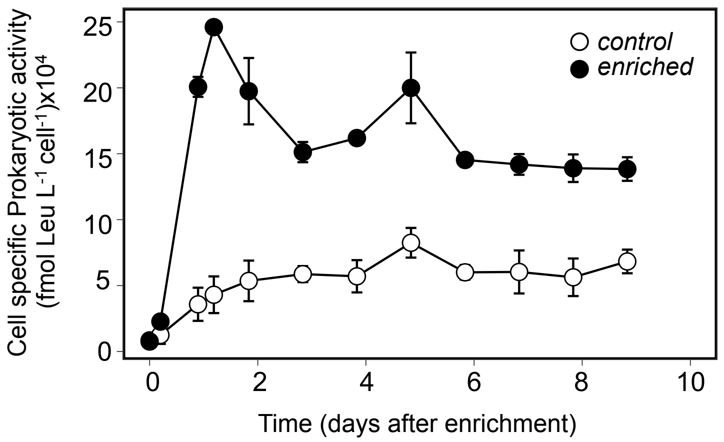

Supplementary figure 1. Temporal dynamics of cell specific heterotrophic prokaryotic production in the control (white dots) and enriched (black dots) treatments of the enrichment experiment. Values represent the average and standard deviation of three replicates.

ß−glucosidase

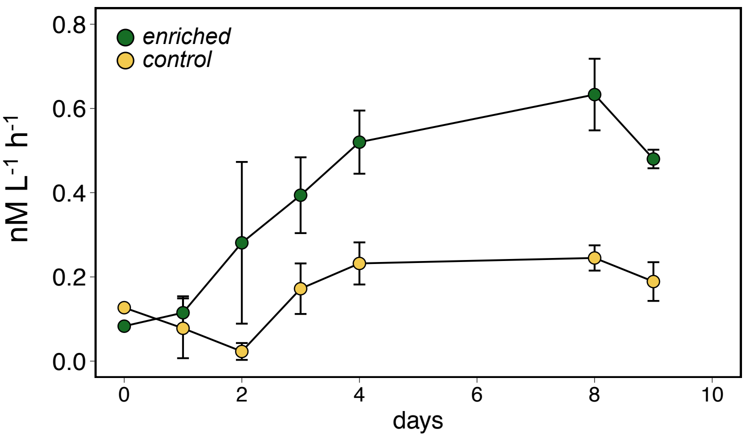

Leu−aminopeptidase

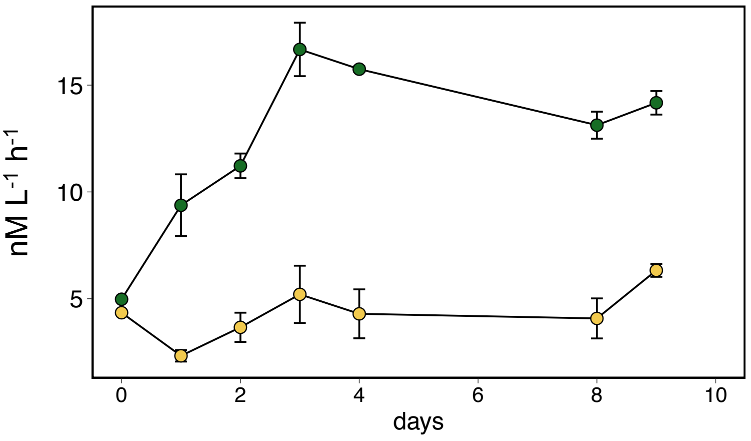

Supplementary figure 2. Temporal dynamics of hydrolytic enzymes involved in the degradation of carbohydrates (ß-glucosidase) and peptides (leu-aminopeptidase) in the control (yellow dots) and enriched (green dots) treatments of the Enrichment experiment. Values represent the average and standard deviation of three replicates.

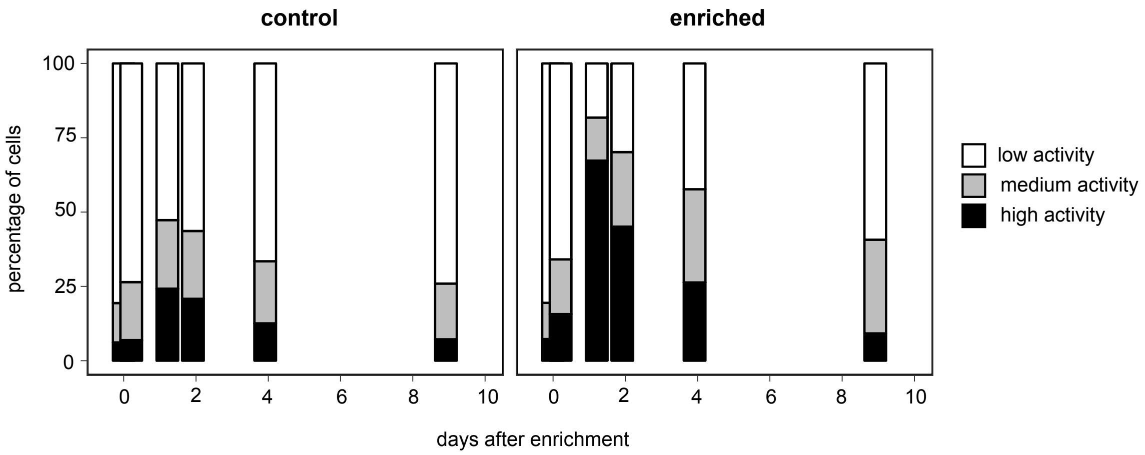

Supplementary figure 3. Percentage of cells with low, medium and high activity in the enrichment experiment, estimated based on the fluorescent intensity of the BONCAT signal of the individual cells. The intensity of the BONCAT+ cells was assessed using the mean gray value, which is the sum of the gray values of all the pixels in the cell divided by the number of pixels. The intensities of individual cells were rank-ordered to obtain the maximum and minimum values and the intensity range was then equally divided into three groups: high intensity (top third), intermediate intensity (middle third) and low intensity cells (bottom third). The percentage of each intensity group within the BONCAT+ cells was then calculated at each time point.

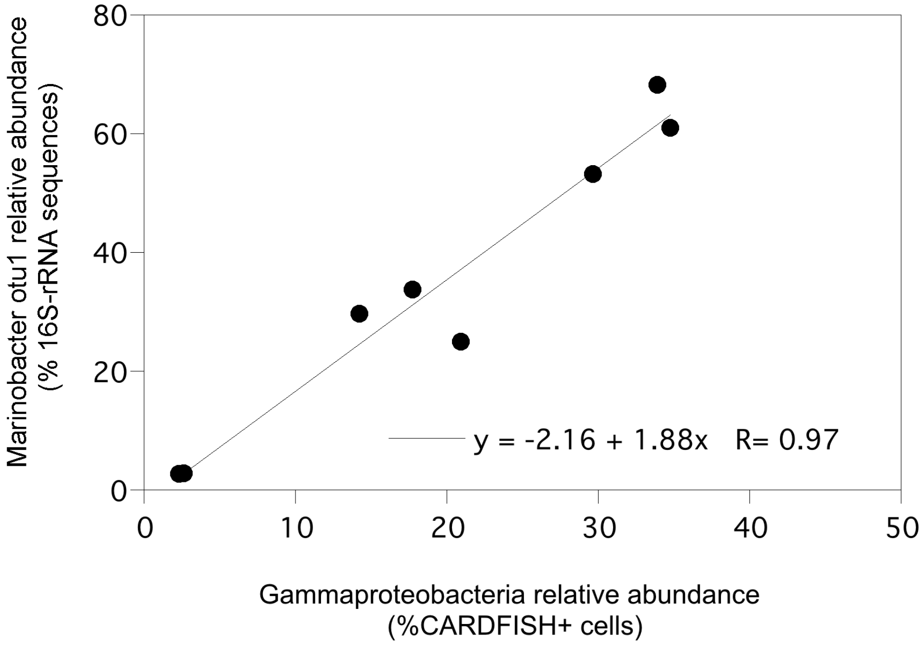

Supplementary figure 4. Comparison of the relative abundance of gammaproteobacterial cells estimated by CARDFISH and *Marinobacter* OTU sequences expressed as percentage of total cells, and reads, respectively.

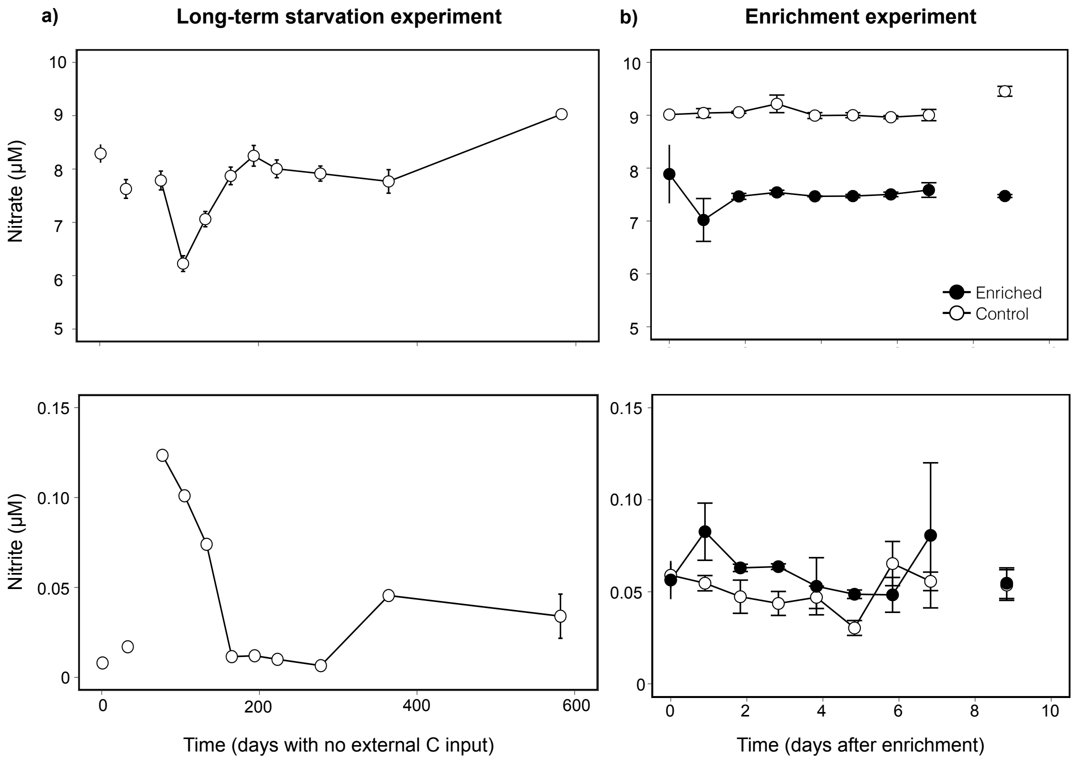

Supplementary figure 5. Dynamics of nitrate and nitrite concentration during a) the long-term starvation experiment and b) the Enrichment experiment. Values represent the average and standard deviation of two technical replicates in the case of the Long-term experiment, and three replicates in the case of the Enrichment experiment. Nitrite displayed a peak coincident with the raise in ammonia oxidizers, but decreased drastically when nitrite oxidizers developed (see Figure 6 in the main text).

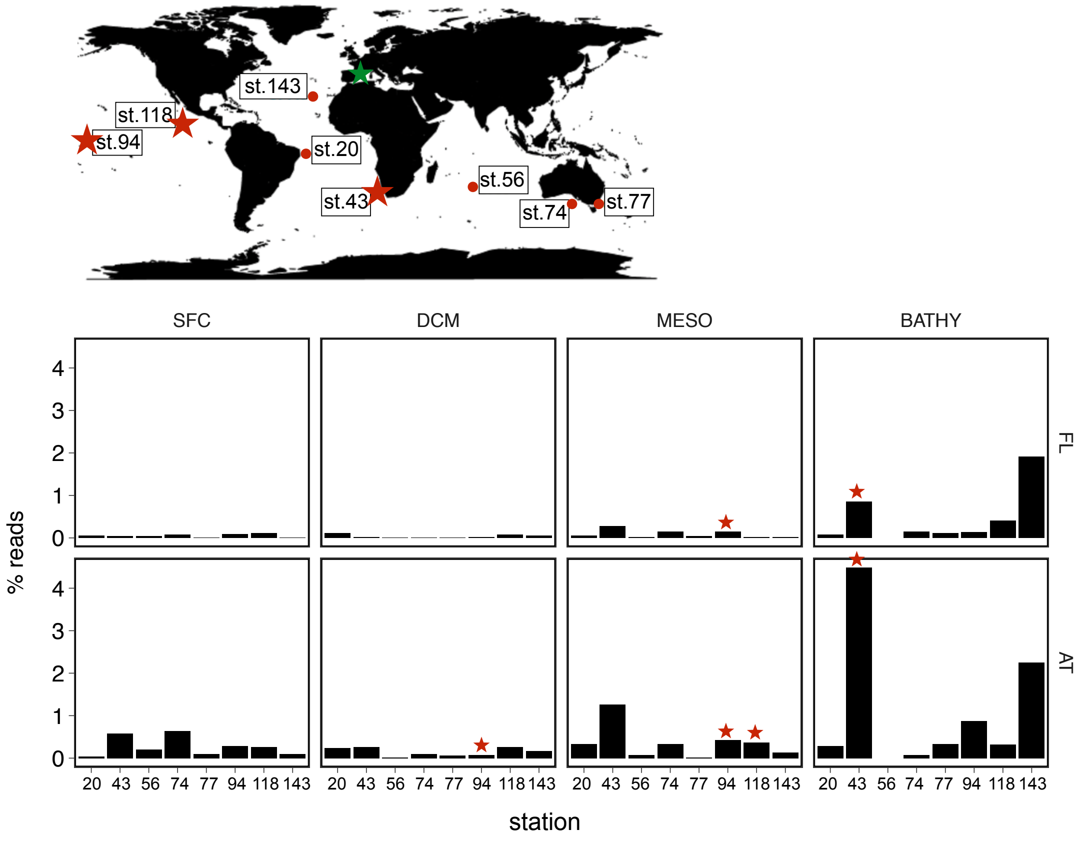

Supplementary figure 6. Contribution of *Marinobacter* OTUs to total sequences in a global oceanic survey (Malaspina 2010, Mestre et al. 2018). Upper panel: free-living communities (0.2-3µm), lower panel: particle associated communities (3-20µm). SFC: surface, DCM: Deep Chlorophyll Maximum depth, Meso: mesopelagic (200-1000m), Bathy: bathypelagic (1000-4000m). Asterisks denote those samples where the *Marinobacter* OTU that dominated in the Enrichment experiment was detected. The green asterisk represents the location where the water for the long-term starvation experiment was collected.

Table S2. Evaluation of the potential contamination of the Enriched treatment with taxa present in the 0.2µm filtered surface seawater used for enrichment. Cell abundances of the *Marinobacter* OTU was calculated using the relationship shown in Figure S4, taking into account the relative abundance of the OTU (%reads) and the total abundance of prokaryotic cells (estimated by flow cytometry). Values are shown as average and standard deviation in parentheses (when possible). Even assuming that all surface derived *Marinobacter* OTU_1 cells were active, they only represented 2.7% of the total pool of Gammaproteobacteria active cells. Growth rate of Gammaproteobacterial cells in the Enriched treatment —estimated by CARDFISH, as in Ferrera *et al.*, (2011)— was 0.11 h^{-1}. Growing at this rate, the surface derived OTU would reach an abundance of 576 cell mL^{-1}, which is two orders of magnitude lower than the actual values. Thus, we can rule out that surface *Marinobacter* cells were the ones driving the response to enrichment.

	**Abundance at t0 (cell mL^{-1})**	**Active cells (cell mL^{-1}) at t0**	**Abundance at 29h (cell mL^{-1})**
Surface-derived *Marinobacter* OTU_1 in the Enriched treatment	21	21	576
Marinobacter OTU_1 in the Enriched treatment	1341 (147)	792 (160)[a]	38981 (115)
Relative contribution of surface derived cells	1.5 %	2.7 %	

[a]Gammaproteobacteria BONCAT+ cells

Supplementary References

Ferrera I, Gasol JM, Sebastián M, Hojerová E, Koblízek M. (2011). Comparison of growth rates of aerobic anoxygenic phototrophic bacteria and other bacterioplankton groups in coastal Mediterranean waters. *Appl Environ Microbiol* **77**:7451–8.

Hoppe H-G. (1983). Significance of exoenzymatic activities in the ecology of brackish water: measurements by means of methylumbelliferyl-substrates . *Mar Ecol Prog Ser* **11**:299–308.

Mestre M, Ruiz-González C, Logares R, Duarte CM, Gasol JM, Sala MM. (2018). Sinking particles promote vertical connectivity in the ocean microbiome. *Proc Natl Acad Sci* **115**:E6799–E6807.

Sala MM, Aparicio FL, Balagué V, Boras JA, Borrull E, Cardelús C, *et al.* (2015). Contrasting effects of ocean acidification on the microbial food web under different trophic conditions. *ICES J Mar Sci J du Cons* **73**:fsv130.
